# Protocols, policies and practices for antimicrobial stewardship in hospitalized patients in least-developed and low-income countries: a systematic review

**DOI:** 10.1186/s13756-023-01335-8

**Published:** 2023-11-23

**Authors:** Grace Wezi Mzumara, Michael Mambiya, Pui-Ying Iroh Tam

**Affiliations:** 1 Malawi Liverpool Wellcome Trust Clinical Research Programme, Blantyre, Malawi; 2 Kamuzu University of Health Sciences, P/Bag 320, Blantyre, Malawi; 3 Liverpool School of Tropical Medicine, Liverpool, UK

**Keywords:** Antimicrobial stewardship, Infection prevention, Healthcare-associated infection, Antibiotic use

## Abstract

**Background:**

We aimed to identify interventions used to implement antimicrobial stewardship practices among hospitalized patients in least-developed countries.

**Methods:**

The research team searched PubMed, EMBASE, and Cochrane Central Register of Controlled Trials for studies of AMS interventions in the least developed and low-income countries, published between 2000 and 2023. Included studies had a population of hospitalized patients of all age groups in least-developed countries, implemented an AMS intervention, and reported its impact on prescription practices, clinical outcomes, or microbiological results. The risk of bias was assessed using the integrated quality criteria for review of multiple study designs. A total of 443 articles were identified, 386 articles were screened, 16 full-text papers were reviewed, and 10 studies were included in the analysis.

**Results:**

The ten studies included three controlled before and after, two qualitative, one controlled interrupted time series, two non-controlled interrupted time series, one quasi-experimental study, and one randomized controlled trial. Three studies implemented either enabling, persuasive, or structural interventions respectively. The rest used bundled strategies, including a combination of persuasive, enabling, structural, and restrictive interventions. Bundled interventions using enabling and persuasive strategies were the most common. These involved creating a prescription guideline, training prescribers on updated methods, and subsequent review and feedback of patient files by members of an AMS team. Improved microbiological surveillance was important to most studies but, sustained improvement in appropriate prescriptions was dependent on enabling or persuasive efforts. Studies noted significant improvements in appropriate prescriptions and savings on the costs of antibiotics. None evaluated the impact of AMS on AMR.

**Conclusion:**

AMS practices generally involve multiple strategies to improve prescription practices. In the setting of least-developed countries, enabling and persuasive interventions are popular AMS measures. However, measured outcomes are heterogeneous, and we suggest that further studies assessing the impact of AMS should report changes in AMR patterns (microbiological outcomes), patient length of stay and mortality (patient outcomes), and changes in prescription practices (prescription outcomes). Reporting on these as outcomes of AMS interventions could make it easier for policymakers to compare which interventions have desirable outcomes that can be generalized to similar settings.

**Supplementary Information:**

The online version contains supplementary material available at 10.1186/s13756-023-01335-8.

## Introduction

Globally, over 5 million people die from diseases or complications of conditions associated with a micro-organism that is resistant to the medication given to treat it [1]. In Sub-Saharan Africa, AMR has been associated with about 16,000 disability-adjusted life years (DALYs) because many infections are resistant to first-line or empirical antibiotics [2–4]. Despite differences in resistance patterns across the world, AMR affects all people because drug-resistant infections spread beyond geographical barriers and are becoming increasingly harder to control [5].

Unfortunately, many low-income countries, lack the resources to identify and monitor antimicrobial resistance patterns [6–9]. In fact, over 40% of African countries have no data on antimicrobial resistance patterns, and, with 78% of antibiotics in low and middle income countries being self-medicated and unregulated, it is increasingly harder monitor antibiotic use [8, 10–12].

Despite these challenges, many studies have shown that it is possible to implement antimicrobial stewardship programs to regulate antibiotic use in hospitals to curb antimicrobial resistance.

Studies that have introduced antimicrobial stewardship interventions have grouped them into enabling, persuasive, structural, and restrictive interventions [13]: enabling AMS programs involve teaching clinical staff about better prescription practices; persuasive AMS programs allow for auditing of prescriptions and the duration of treatment and constructively discussing these with the prescriber; structural programs involve the judicious use of diagnostics to ensure antibiotics are prescribed for the right organism; and restrictive interventions involve hospital or regional level policies that restrict availability of antibiotics to specific groups, prescribers or organisms [13].

Systematic reviews on antimicrobial stewardship have highlighted methods to curb antimicrobial resistance in different settings globally. Among low and middle income countries, clinical decision making tools were found to be efficient methods of improving prescription practices [10].

Other systematic reviews on interventions to improve antimicrobial prescribing in hospital inpatients found that; antimicrobial stewardship programs decreased the duration of treatment and reduced length of stay, AMS improved adherence to prescription recommendations, restrictive interventions were associated with increased compliance, and that enabling interventions of audit and feedback and were highly effective [14–17]. In these studies, reduction in duration of treatment was not associated with an increase in mortality and this represents an economic incentive to control antibiotic use in resource constrained settings.

However, the gap in these studies is that systematic reviews on low- and middle-income countries focus on middle income countries of China, Indonesia and south Africa, which represent middle income countries [9, 10, 14, 17].

In this systematic review, we aim to identify the protocols, policies and practices used for antimicrobial stewardship in hospitalised patients of all ages in the least developed and low-income countries and to provide comprehensive information on how AMS can be carried out in these settings.

## Methods

We conducted a systematic review of AMS practices in least developed and low-income countries. The protocol for this review was registered and published with the international prospective register of systematic reviews (PROSPERO): CRD42020210634 [18].

### Search strategy

**Fig. 1:**
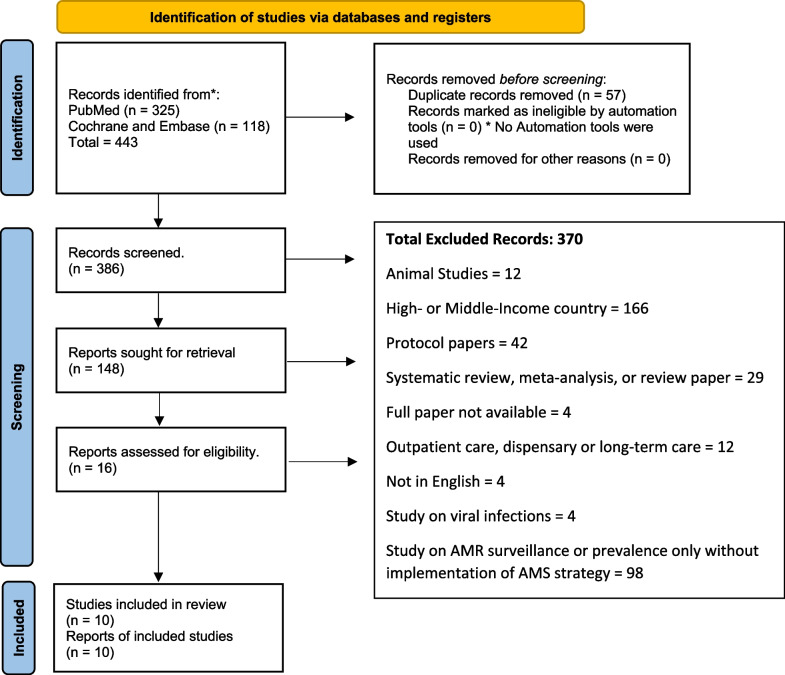
Antimicrobial Stewardship in Least-Developed and Low-Income Countries – Prisma Flow Diagram. *Consider, if feasible to do so, reporting the number of records identified from each database or register searched (rather than the total number across all databases/registers). **If automation tools were used, indicate how many records were excluded by a human and how many were excluded by automation tools. *From:* Page MJ, McKenzie JE, Bossuyt PM, Boutron I, Hoffmann TC, Mulrow CD, et al. The PRISMA 2020 statement: an updated guideline for reporting systematic reviews. BMJ 2021;372:n71. 10.1136/bmj.n71. For more information, visit: http://www.prisma-statement.org/

Between November 2020 and 11th September 2023, two reviewers (GM and MM) independently identified studied studies by searching PubMed, EMBASE, and Cochrane Central Register of Controlled Trials (CENTRAL). Records were screened for the inclusion and exclusion criteria at abstract, and full-text review as demonstrated in the Prisma Flow Chart in Fig. 1. Search terms and databases used are described in Additional file 1.

### Inclusion and exclusion criteria

We included original research from studies that implemented AMS interventions in hospitalized patients published in English between 2000 and 2023, conducted in a least developed or low-income country based on the Development Assistance Committee (DAC) classification [19]. We extracted data from randomized controlled trials, controlled before and after trials, interrupted time series studies, cohort, and qualitative studies. Our study population was hospitalized children and adults of all ages with bacterial infections, including patients with surgical and obstetric conditions.

We included structural, enabling, persuasive, and restrictive interventions and searched for behavioral, clinical, and microbiological outcomes. Structural interventions are those where the intervention was technology to guide antibiotic treatment [13]. These include new laboratory equipment, mobile phone applications or algorithms used to discern bacterial infections and their levels of antibiotic susceptibility. Persuasive interventions mostly involve reviewing prescriptions and providing feedback to the prescriber on how they could improve the appropriateness of the drug chosen and the duration of treatment [13]. Enabling interventions implement ways to educate prescribers but do not review their prescriptions. This can be done through treatment guidelines, classes or seminars on antimicrobial resistance and prescription guidelines [13]. Restrictive interventions are those that require approval for certain antibiotics. This can be from the pharmacy level, infectious disease specialist level or AMS team level. We also included studies which used a combination of any of these types of interventions.

The exclusion criteria were interventions on patients in communities, pharmacies, and dispensaries. We also excluded studies that only reported on prevalence of AMR, hospital related infections or prescription practices without implementing an intervention. We documented whether AMR was an endpoint and how that was measured.

The studies were assessed for microbiological, clinical, behavioral, and prescriptive outcomes. Microbiological outcomes include changes in resistance patterns. Clinical outcomes included changes in length of stay, days of treatment, hospital related infections (including surgical site infections). Prescriptive outcomes are changes in prescription practices. Other outcomes that were identified from the studies include the costs of treatment.

We adapted the Cochrane collaboration data collection form to extract data and used the 2020 Preferred Reporting Items for Systematic Reviews and Meta-Analyses (PRISMA) guidelines to present our findings [20–22]. Reviewers independently used the Integrated Quality Criteria for Review of Multiple Study Designs (ICROMS) to assess for risk of bias and consolidated findings with a third reviewer [23]. The Prisma 2020 checklist for this study is presented in Additional file 2. No automation materials were used during the entire process of this study.

## Results

We identified 443 articles, and after 57 duplicates were removed, we screened 386 records (Fig. 1). The included studies were published between 2015 and 2022, and were from Malawi, Tanzania, Nepal, Cambodia, Ethiopia, Uganda, Liberia, Bangladesh and Mali and involved a total of 3295 patients (Table 1). Two studies focused on obstetric patients, two on pediatric patients only, three on adults over 15 years, and three on patients of all ages. Among the study designs included were two were controlled before and after, two were qualitative, one was a controlled interrupted time series, one non-controlled interrupted time series, and one non-controlled before and after study. Enabling interventions were the most common type of intervention used and were present in eight of the ten studies. Three studies used one type of intervention; enabling, structural or persuasive respectively; other studies used bundled interventions involving at least two types of interventions. All outcomes from the included studies are reported in Table 1. Due to variation in how outcomes were reported, a meta-analysis was not done.

**Table 1:** Findings of AMS interventions with their clinical, microbiological and prescription outcomes

Author	Country and setting	Type of study and duration of data collection	Population	AMS development	Intervention type	Outcome description
Hall et al. [34]	Tanzania, Tertiary Care Hospital	Qualitative Study January 2017–December 2018	Adult medicine and paediatric wards	1. Baseline Clinician Survey on knowledge of AMS/AMR 2. Chart review of antimicrobial prescribing practices 3. Baseline audit of bacterial species and Antimicrobial Resistance Patterns 4. Local pharmacy survey Creating an AMS guidebook focused on the common organisms isolated in that location. Guidebook covering empirical antibiotic coverage based on resistance patterns, availability of the antibiotics and the national guidelines. Guidebook to be electronically distributed	Enabling	No outcome
Joshi et al. [27]	Nepal, Tertiary Care Hospital	Controlled Interrupted Time Series August 2016–August 2017	Adults over 15 years in medical, surgical and obstetric wards	1. Laboratory surveillance for baseline 2. Antibiotic prescribing guidelines based on common infections and drug availability 3. Post-prescription review and feedback adaptation led by physician champions in study wards, and stewardship training 4. Post-prescription review and feedback evaluation	Bundle: Persuasive and Enabling	1. Behavioral outcomes: (A) Recommendations by physician champions were most followed and in the medicine ward 2. Clinical outcomes: (A) DOT per 1,000 PD increased from 761 to 823 (*P* < 0.001) for intravenous therapy, and DOT for oral therapy increased from 302 to 390 (*P* < 0.001) Cephalosporin use decreased from 420 DOT/1000 PD to 344 (*P* < 0.0001) and aminoglycoside decreased from 138 DOT/1000 PD to 95 (*P* < 0.001) (B) No significant difference in length of stay
Gebretekle et al. [28]	Ethiopia, Tertiary care hospital	Controlled Interrupted Time Series November 17–January 2019	Adult medicine and paediatric wards	1. Adapting antimicrobial prescribing guidelines according to local common indications for antimicrobial prescription 2. Institutional microbiogram from laboratory data 3. Training pharmacists and giving AMR information sessions for clinicians 4. Pharmacists from the AMS issue a recommendation and followed up to assess acceptance or non-acceptance of the intervention	Bundle (Enabling and Persuasive)	1. Behavioral—Reduction in antibiotic prescription without documented source during post-intervention phase (25% vs. 16%) 2. Clinical: (A) Reduction in hospital acquired Infection (78% vs. 66%) (B) Days of Therapy: Days of antibiotic therapy increased: from 8.7 ± 6.9 days during the intervention, to 12.8 ± 11.7 days post intervention for all antibiotics. A twofold increase in DOT mean DOT of 754 ± 99.8/1000 patient-days in the intervention phase to 1549 ± 175.2/1000 patient-days during the following 5 months (C) Length of Stay in hospital increased from 19.8 ± 12.0 days to 24.1 ± 13.9 (20% increase in duration, *p* < 0.001) (D) All-cause mortality increased from 6.9% during the intervention to 14.7% post–intervention *p* < 0.01
Ackers et al. [31]	Uganda, Tertiary Care Hospital	Qualitative Study January 2019–January 2020	Obstetric patients	1. Four-month baseline data on drug orders and supplies from national medical stores and dispensing log 2. Introduction of swabbing post-caesarean wounds for laboratory diagnosis of infection (culture and sensitivity) and wound cleaning (Infection prevention) 3. Laboratory data on samples from post-natal ward 4. Survey on Perceptions of the impact and effectiveness of the AMS program 5. New policy to restrict high-end antibiotics culture and sensitivity indication	Structural and restrictive	1. Clinical: Increase in number and frequency of patient’s wounds being cleaned daily with culture and sensitivity from 0 to 95% of all suspected sepsis patients having culture and sensitivity 2. Behavioral: (A) Better patient care for women with septic wounds (B) Better collaboration among pharmacists, nurses and physicians in caring for patients 3. Procurement: Pharmacists became judicious in ordering of antibiotics using the information from the laboratory culture and sensitivity testing
Hearn et al. [29]	Cambodia, Hospital	Controlled Before and After January15–December 2015	Children	1. Pre-existing hospital antimicrobial guidelines were updated and converted into a free smartphone app 2. Pre-existing Point prevalence survey supported by a clinical microbiologist	Bundle: Enabling and Persuasive	1. Clinical: (A) Decrease in HAI incidence during surveillance period (B) Median LOS for HAI cases increased for non-HAI controls: 25 days IQR 12–37 vs 5 days IQR 3–9 2. Microbiological: (A) 81% isolates were gram negative organisms. (B) Third generation cephalosporins were ineffective against 75% of clinical isolates 3. Behavioral: Increased use of guidelines in prescribing. 75.4% of antimicrobial prescriptions were appropriate
Gentilotti et al. [32]	Tanzania, Hospital	Controlled Before and After August 2013–August 2015	Obstetric Patients	1. Training in Antimicrobial resistance and appropriate prescribing 2. Developing an AMS multidisciplinary team to create and monitor AMS policies	Bundle: Enabling and Persuasive	1. Clinical: 65% reduction in CS SSIs 2. Microbiological: Lower SSI with Pfannenstiel incision (OR 0.288; 95% CI 0.197–0.420; *p* < 0.001) 2. Post-op antibiotic administration decreased post intervention 3. Behavioral—Increased use of skin disinfection. Improved adherence to standard operating procedures in the theatre 4. Microbiological: (A) Increased rate of microbiologically confirmed SSI was higher in post int group (OR 2.534; 95% CI 1.435–4.475; *p* = 0.001). (B) Decrease in prevalence of gram-positive SSI significantly decreased (OR 0.263; 95% CI 0.126–0.548; *p* < 0.001) (C) Reduced MRSA prevalence (79–21.4% (OR 0.072; 95% CI 0.016–0.314; *p* < 0.001) (D) Increase in the prevalence gram-negative SSI (including *Klebsiella* and *Pseudomonas*.) (OR 3.800; 95% CI 1.822–7.926; *p* < 0.001)
Lester et al. [30]	Malawi, Tertiary hospital	Controlled before and after, Mixed methods study	Adults only	Pre-implementation prescribing survey Antibiotic treatment guideline* booklet and app Post-implementation prescribing surveys Point-prevalence surveys with feedback to prescribers	Bundle, enabling and Persuasive	1. Clinical: 26.5% reduction in third generation cephalosporin use 2. Reduced treatment time for ceftriaxone from 5 to 4 days 3. No difference in mortality or length of stay
Nauriyal et al. [25]	Nepal, Three Hospitals	Controlled Before and After January 2018–January 2019	Adults with a burn or a wound (> 15 years)	1. Post prescription review and feedback program 2. Baseline and post-intervention chart review	Enabling	1. Decrease in days of therapy for penicillin, aminoglycosides, and cephalosporin 2. Decrease in days of therapy for intravenous antibiotics 3. Improved appropriate prescription, de-escalation, documentation, and adherence to treatment guidelines
Alabi et al. [33]	Liberia	Non-controlled before and after December 2019–December 2021	Adults and children	1. Microbiology laboratory for culture and sensitivity 2. Multidisciplinary AMS team 3. Treatment guideline, prescriber training and three weekly AMS ward rounds	Bundle: Enabling and Structural	1. Improved appropriate prescription 2. Challenges in adoption of prescribing guidelines: 39% in appropriate antibiotic chose, 63.5% had incorrect dose and 69% had incorrect duration 3. Use of microbiology laboratory for 79.7% of patients with infectious disease
Nelson et al. [26]	Bangladesh and Mali	Randomized cross over study. January 2021–November 2021	Children under 5 years	1. Development of diarrheal aetiology prediction algorithm 2. Physicians randomised to intervention and control arm 3. Study done at three sites in Bangladesh and 4 sites in Mali	Structural	1. No statistically significant difference in proportion of children prescribed antibiotics in intervention and control arm 2. No known adverse events from DEP tool

### Single interventions

#### Enabling intervention

Clinicians and researchers at a referral hospital in Mbeya, Tanzania created an antimicrobial prescription guidebook to ensure good prescribing practices among clinicians between 2017 and 2019 [24]. Before creating the book, they conducted comprehensive baseline studies to understand the state of AMR and prescribing practices in their area. They conducted clinician surveys to understand the knowledge of AMR and AMS at the hospital, chart reviews to assess prescribing patterns, culture and sensitivity reviews to understand resistance patterns at the hospital, and pharmacy surveys to assess the availability of over-the-counter antibiotics in the region. The study found that 7 of 38 junior clinicians did not know about AMS, 50% of empirical treatments were not aligned to national guidelines, about 66% of in-hospital antimicrobial courses were not completed, and, there was high resistance of *Escherichia coli* to cotrimoxazole [24]. Using this information, the antimicrobial prescription book was made available physically and electronically to enable access during clinical work. The impact of these interventions on prescribing practices and AMR was not measured.

#### Persuasive intervention

Nauriyal et al. [25] conducted a post-prescription review and feedback program in three hospitals to improve antibiotic prescribing for in patient adults (over 15 years) with wounds or burns in Nepal. Infectious disease specialists trained physician champions and updated the antibiotic guidebook to cover treatment for wounds and burns. Baseline chart reviews were done for 6 months (January 2018–June 2018), followed by a one-month (July 2018) implementation, and a six-month post-intervention chart review phase (August 2–18 to January 2019). The result of this intervention was that days of treatment with intravenous antibiotics were reduced (10.1 days at baseline and 8.8 days post-intervention, t = 3.56; *p* < 0.001) [25]. There was significant improvement in prescription practices through appropriate prescriptions, improved documentation, de-escalation, and adherence to prescribing guidelines.

#### Structural intervention

A smart-phone based diarrheal etiology prediction tool (DEP) was developed to help prescribers differentiate between viral and bacterial causes of diarrhea in children [26]. A randomized cross over study was then conducted to determine if this reduced antibiotic prescriptions in children under five in Bangladesh and Mali. There was no statistically significant difference in antibiotic prescription between children with the DEP arm and the control arm (RD − 4.2%, 95% CI − 10.7 to 1.0%) [26].

### Bundled interventions

#### Persuasive and enabling interventions

At a hospital in Nepal, a joint persuasive and enabling intervention involved creating an antimicrobial prescribing guideline and post-prescription review and feedback [27]. Pre-intervention screening was done for 221 patient charts which revealed that 31.6% of antibiotic prescriptions were unjustified [27]. The guideline included empirical and definitive antibiotic therapy and was based on a hospital antibiogram created using hospital antibiotic sensitivities. After the intervention, 230 charts were reviewed and 78% of prescribers followed recommendations made by prescription champions to improve prescriptions, de-escalation and documentation of antibiotic use [27].

In Ethiopia, an audit feedback intervention that recruited 1264 patients over 10 months was used for prescriptions for sepsis, febrile neutropenia, and hospital- and community-acquired pneumonia, in general medicine and pediatric wards [28]. The enabling intervention was antimicrobial prescribing guidelines made into an accessible app, and the persuasive intervention was reviews of the treatment of 25% of admitted patients. Following the discussion of these cases, the antibiotic therapy was either stopped, changed, adjusted for the duration of treatment or a consult with an infectious disease specialist was recommended [28]. After the intervention, there was an increase in days of antibiotic treatment from 8.7 ± 6.9 days to 12.8 ± 11.7 days, and the mean days of therapy per 1000 patient days (DOT/1000) doubled from 754 ± 99.8/1000 patient days to 1549 ± 175.2/1000 patient days [28]. There was a 20% increase in length of stay from 19.8 ± 12.0 days during the intervention period, to 24.1 ± 13.9 days after the intervention (*p* < 0.001) and an increase in all-cause mortality from 6.9 to 14.7% post-intervention (*p* < 0.01) [28]. The authors attributed these findings to an increased knowledge of second-line antibiotics, and their increased use leading to longer hospital stays. The increase in all-cause mortality was attributed to the absence of infectious disease specialist involvement during the intervention. These specialists likely provided critical treatment advice to reduce mortality during the intervention [28].

In Cambodia, bundled AMS interventions were implemented in a paediatric hospital [29]. The enabling intervention was transforming antimicrobial prescription guidelines to a mobile phone app and the persuasive intervention was using point prevalence surveys to inform antibiotic prescriptions [29]. The most common hospital-acquired infections were hospital-acquired pneumonia, ventilator-associated pneumonia, lower and upper respiratory tract infection, and necrotizing enterocolitis in neonates [29]. This study noted a 75% increase in appropriate antibiotic prescriptions and a downward trend in mortality after antimicrobial surveillance started.

In Malawi, a quasi-experimental study was done to reduce cephalosporin use in adults at a tertiary hospital [30]. A multidisciplinary team supervised the intervention which involved a baseline prescription survey, creating an antibiotic treatment guideline available as a mobile application, a post-implementation survey, and prescription feedback given by infectious disease specialists. Blood cultures were done to monitor sensitivity patterns and *Salmonella* Typhi, *Salmonella* Typhimurium and *Klebsiella pneumoniae* were the most isolated pathogens. The outcome was a 27% reduction in the use of third-generation cephalosporins and a 9% decline in prescriptions that were made without an indicated focus of infection among 203 patient charts that were screened [30]. Although there was no difference in mortality and length of stay, the intervention was estimated to have saved about $15,000 in the costs of antibiotics [30].

#### Structural and restrictive interventions

A study in Uganda that used structural and restrictive interventions targeting surgical site infections from cesarean sections highlighted the value of wound care as part of IPC to prevent AMR [31]. The structural intervention was swabbing post-cesarean section wounds for culture and sensitivity to guide antibiotic prescription. Following a year of the intervention, 90% of patients suspected to have sepsis had culture and sensitivity performed on wounds from their wounds [31]. This was attributed to a new policy that restricted the prescription of high-end antibiotics to those necessitated by culture and sensitivity. Apart from clinical outcomes, the study reported improved care of surgical wounds and collaboration between clinicians, nurses, laboratory staff, and pharmacists as important outcomes which contributed to AMS in the post-natal ward [31]. Another outcome was that the procurement of antibiotics changed to reflect reported antibiotic sensitivities [31].

#### Structural and enabling interventions

In Tanzania a tertiary hospital combined IPC and AMS programs for post-caesarean section and surgical site infections for 1377 patients [32]. The intervention involved appropriate pre and post-operative antibiotic administration, infection prevention measures during surgery, and training in AMS and IPC [32]. The result was a decrease in overall surgical site infections during the post-intervention survey, with a decrease in gram-positive infections (OR 0.263; 95% CI 0.126–0.548; *p* < 0.001) and a decrease in the prevalence of methicillin-resistant *S. aureus* (MRSA) from 79 to 21.4% (OR 0.072; 95% CI 0.016–0.314; *p* < 0.001) [32].

Researchers in Liberia used structural and enabling interventions to introduce AMS to three hospitals [33]. The structural intervention was establishing a microbiology laboratory for culture and sensitivity, available to the three hospitals. The enabling intervention was the creation of a multidisciplinary AMS team to create prescription guidelines, and train prescribers during AMS ward rounds that occurred three times a week. Despite high use of empirical antibiotics and challenges in adopting prescribing practices, the structural changes meant that a blood culture was conducted for 79.7% of patients suspected to have an infectious disease [33].

## Resources for antimicrobial stewardship practices

Antimicrobial surveillance was key to formulating locally relevant AMS protocols. In Tanzania, surveillance revealed high *E. coli* resistance, particularly against trimethoprim-sulfamethoxazole and penicillin’s [32, 34]. In Nepal, the majority of isolates were *E. coli* (42%), and *Klebsiella* spp. (16%) and were highly resistant to penicillin’s and third-generation cephalosporins [27]. Third-generation cephalosporins were antibiotics with high resistance to 75% of clinical isolates [29].

Three studies described creating a multidisciplinary team as a key feature of the AMS intervention. Hall et al. described a team of physicians and nurses selected to monitor the implementation of AMS practices [24]. In Joshi et al. physician champions, who were doctors in medical, surgical, and obstetric specialties, were trained to monitor prescription patterns within their respective wards [27]. In one study in Ethiopia, pharmacists led the surveillance and production of AMS protocols together with an infectious disease specialist [28]. Our findings complement a systematic review which showed that pharmacist led intervention improved adherence to prescription guidelines [18].

### Risk of bias assessment

Table 2 shows the risk of bias scoring according to the Integrated Quality Criteria for review of Multiple Studies (ICROMS). Two studies did not meet the ICROMS criteria. One qualitative study did not demonstrate the outcomes of the study, and one non-controlled before and after did not sufficiently describe its baseline assessment group [24, 33].

**Table 2:** Integrated quality criteria for review of multiple studies (ICROMS)

Dimension	Specific criteria	Ackers et al. [31]	Joshi et al. [27]	Gebretekle et al. [28]	Hall et al. [24]	Hearn et al. [29]	Gentilotti et al. [32]	Lester et al. [30]	Nauriyal et al. [25]	Alabi et al. [33]	Nelson et al. [26]
Study type		Qualitative	ITS	N-CITS	Qualitative	CBA	CBA	NCBA (Quasi experimental)	CBA	NCBA	RCT
Clear aims and justification	Clear statement of the aims of the area of research	YES^1^	YES	Yes	YES	YES	YES	YES	YES	YES	YES
	Rationale for number of pre- and post-intervention points or adequate baseline measurement		2	Yes				YES		NO	
	Explanation for lack of control group			2				2		1	
	Appropriateness of qualitative methodology	2			2						
	Appropriate study design	YES			YES						
Managing bias in sampling or between groups	Sequence generation										YES
	Allocation concealment										YES
	Justification for sample choice			Yes				YES		YES	
	Intervention and control group selection designed to protect against systematic difference/detection bias					YES	YES		YES		
	Comparability of groups										
	Sampling and recruitment	Yes (convenience sampling)			YES (convenience sampling)						
Managing bias in outcome measurements and blinding	Blinding										YES
	Baseline management—protection against selection bias					YES	YES		YES		
	Protection against contamination					YES	YES		YES		
	Protection against secular changes		YES (post intervention survey was during another season)								
	Protection against detection bias: Blinded assessment of primary outcomes		2	2		2	2	2	2	1	2
	Reliable outcome measures	0	2	2	2	2	1	2	2	2	2
	Comparability of outcomes										
Managing bias in follow-up	Follow-up of subjects (protection against exclusion bias)										2
	Follow-up of patients or episodes of care										2
	Incomplete outcome data addressed	0	2	2	2	2	1	2	2		2
Managing bias in other study aspects	Protection against detection bias: Intervention unlikely to affect data collection		2	2		2	2	2	2		2
	Protection against information bias										
	Data collection appropriate to address research aims	1			2						
	Attempts to mitigate effects of no control			YES				YES			
Analytical rigour	Sufficient data points to enable reliable statistical inference		YES								
	Shaping of intervention effect specified		2								
	Analysis of sufficiently rigorous/free from bias	1	2	1	2	2	2	2	2	2	2
Managing bias in reporting/ethical considerations	Free of selective outcome reporting	0	2	2	2	2	2	2	2	2	2
	Limitations addressed	2	2	2	2	2	2	2	2	2	2
	Conclusions clear and justified	2	2	2	2	2	2	2	2	2	2
	Free of other bias	1	2	2	1	2	2	2	2	2	2
	Ethics issues addressed	2	2	2	2	2	2	2	2	2	2
Total score		11	24	21	19	20	18	22	22	20	24

^1^YES = Mandatory criteria met, 1 = Score for unclear description, 2 = Score for satisfactory description

## Discussion

This systematic review of AMS practices in least developed and low-income countries showed that only a handful of countries have evaluated AMS in their settings; and that of these practices, bundled interventions including an enabling approach are the most studied. Enabling interventions, where teaching tools are used to guide the choice of antibiotic prescribed, were the most common. Microbiological surveillance revealed the presence of hospital acquired infections and resistance patterns that needed to be addressed and was an important tool to provide feedback to clinicians and policymakers at hospital level. None of the studies evaluated the impact of AMS on AMR.

These results showed that multidisciplinary involvement reinforced judicious prescription practices among clinicians, when pharmacists and nurses were involved in developing AMS protocols. Feedback on prescription practices can occur at ward, laboratory, and pharmacy levels and can be provided by nurses, clinical officers, pharmacists, or doctors [35, 36]. Pharmacy-led interventions, when pharmacists participated in patient care during ward rounds and in formulating AMS protocols, have demonstrated a reduction in inappropriate prescribing and better adherence to AMS protocols [36]. This demonstrates the importance of multi-disciplinary AMS teams to improve patient care and can be of particular benefit in settings with staff shortages [37, 38]. Dedicated AMS team members, like “AMS champions”, at critical levels of health care in low-resource settings can be used to advocate for, and implement measures that can improve the practice of AMS protocols in these settings [27, 39].

Several studies incorporated IPC measures into their AMS programs to reduce hospital-acquired infections [29, 31]. Incorporating IPC in surgical care is a recurring theme in curbing AMR that resulted in reduced surgical site infections and MRSA infections [32, 40]. In surgical settings, enabling AMS interventions were combined with structural changes to patient management, leading to improved wound care and reduced recurrent hospital infections. Similarly, studies in Kenya, Uganda, Zambia and South Africa used antiseptic pre- and post-operative antiseptics for patients to decrease the likelihood of post-operative surgical site infections [41, 42]. The COVID-19 pandemic demonstrated how important IPC measures are for both medical and surgical patients, and should form an important part of any AMS program [37, 43, 44].

The included studies created their own AMS protocols based on a biogram from their own surveillance data to understand local resistance patterns [24, 27, 31]. Although microbiology is important so that hospital-specific organisms can be targeted, many low-income settings lack the laboratory infrastructure to support this [45] and more than 40% of African countries have no data on AMR [8]. The WHO Access, Watch, and Reserve (AWaRe) protocol guides antibiotic prescribing according to the risk of resistance, and this can be used to develop hospital-level AMS protocols [46], and be incorporated into mobile apps like those used in Ethiopia and Cambodia [28, 29]. In the absence of a biogram, this could be an accessible, evidence-based way to guide prescriptions.

Although all studies reported positive outcomes of varying degrees, after applying AMS strategies, it is difficult to extrapolate the impact on large populations because of the varying nature of the interventions. A 2022 systematic review involving high and middle-income countries found that AMS interventions reduced the length of stay, and days of antibiotic treatment [17]. In resource-limited settings, the lack of data on improvements in mortality and length of hospital stay reflect the complex nature of the diseases that occur [24, 30]. To make studies comparable and generalizable to large populations, outcomes from AMS interventions should not be limited to judicious antimicrobial use alone, but could also include clinical, microbiological, and cost-effectiveness data [13].

Incorporating feedback on prescriptions was a key mechanism in AMS practices. In all settings, providing constant feedback to prescribers about the appropriateness of their prescriptions was important to achieve sustained appropriate prescribing and improvement of clinical outcomes. In Nepal, 78% of prescription recommendations were adopted and feedback had a positive impact on patient outcomes [27]. While there were reported decreases in hospital-acquired infections and surgical site infections in Cambodia and Tanzania [29, 32], studies in Nepal and Ethiopia reported an increase in days of antibiotic use and hospital stay after prescription training [27, 28]. This indicates the need to create mechanisms for constant feedback on prescription practices at all levels of health care. This also shows that even without microbiological surveillance, reporting on new infections and complications might inform health institutions on the effect of AMS practices.

At national and regional levels, feedback mechanisms should include reporting AMR patterns as the lack of these data has impeded the development of interventions to address AMR [8]. The WHO Global Antimicrobial resistance and use Surveillance System (GLASS) can be used by researchers and policymakers to monitor AMR trends and guide AMS protocols at national policy level [1].

This systematic review was limited by the paucity of data on AMS in least developed and low-income countries. The included studies did not use standardized metrics or report on patient outcomes consistently and few provided quantitative data on outcomes, including AMR, as has been discussed extensively in other reviews [10, 42]. Therefore, we were unable to quantify the impact of AMS protocols on the populations studied. To address this in future studies, we suggest standardized reporting of outcomes of AMS interventions. This means reporting on a minimum set of outcomes such as baseline and follow-up patterns of AMR (microbiological outcomes), reporting clinical outcomes reflected by patients’ baseline and follow-up length of stay and mortality rates (patient outcomes), and reporting changes in prescription practices (prescription outcomes). This would make it easier to compare results from different studies and help decision-makers in health institutions to decide on which interventions would benefit their population.

## Conclusion

In conclusion, AMS has been demonstrated to be effective in high-income countries in addressing AMR, but we identified only a handful of studies evaluating AMS interventions in least developed and low-income countries. None evaluated the impact of AMS on AMR. Clinicians and policymakers looking to implement AMS interventions in resource-constrained settings could consider firstly, creating multidisciplinary AMS teams incorporating infection prevention strategies in clinical wards and surgical theatres. Secondly, where possible, it is important to have antimicrobial surveillance. This could be done continuously where resources are available or at pre-specified time points to guide formulation of AMR guidelines which should be available physically and electronically. Regular and constructive feedback from the health care team, nurses and pharmacists included, could improve the performance of the clinical team. Information on clinical complications and the cost of changing antibiotics could be included in reports of AMR patterns. Lastly, we suggest that studies on AMS interventions should have standardized reporting on microbiological, clinical, and prescription practice outcomes. This would make results comparable and help policymakers to decide on which interventions would suit their setting.

### Supplementary Information

**Additional file 1.** Search Strategy.

**Additional file 2.** PRISMA 2020 Checklist.

## Data Availability

All information used for data extraction and quality analysis is available upon request to the corresponding author.
