# Supplementary material for: Protocols, policies and practices for antimicrobial stewardship in hospitalized patients in least-developed and low-income countries: a systematic review
Source: Antimicrob Resist Infect Control. 2023 Nov 23;12:131. doi: 10.1186/s13756-023-01335-8 (PMC10666353; doi:10.1186/s13756-023-01335-8)
Supplement: Supplementary file 1 — Additional file 1. Search Strategy. [file 13756_2023_1335_MOESM1_ESM.docx]

**Protocols, Policies and practices for antimicrobial stewardship in hospitalized patients in least-developed and low-income countries – a systematic review**

**Search Strategy**

1. **Range of publication dates: 2000 to 2023**
2. **Search terms according to PICO Guideline**

| PICO | Search Terms |
| --- | --- |
| P | Least developed countries, pharmacist, doctors, nurses, hospitalized patients |
| I | Antimicrobial stewardship programs or antimicrobial stewardship program, infection prevention, surgical site care |
| C | Patient groups who did not receive the intervention, Randomised Controlled Trial, Before and After, Time interrupted series |
| O | Mortality, prescription practices, hospital stay, days of therapy |

1. **Database: Pubmed**

**Database search as of 12^th^ August 2023**

("antimicrobial stewardship"[MeSH Terms] OR ("antimicrobial"[All Fields] AND "stewardship"[All Fields]) OR "antimicrobial stewardship"[All Fields]) AND ("developing countries"[MeSH Terms] OR ("developing"[All Fields] AND "countries"[All Fields]) OR "developing countries"[All Fields] OR ("low"[All Fields] AND "income"[All Fields] AND "countries"[All Fields]) OR "low income countries"[All Fields]) AND (("5"[All Fields] AND ("afghanistan"[MeSH Terms] OR "afghanistan"[All Fields] OR "afghanistan s"[All Fields])) OR ("angola"[MeSH Terms] OR "angola"[All Fields] OR "angola s"[All Fields]) OR ("bangladesh"[MeSH Terms] OR "bangladesh"[All Fields] OR "bangladesh s"[All Fields]) OR ("benin"[MeSH Terms] OR "benin"[All Fields] OR "benin s"[All Fields]) OR ("bhutan"[MeSH Terms] OR "bhutan"[All Fields] OR "bhutan s"[All Fields]) OR ("burkina faso"[MeSH Terms] OR ("burkina"[All Fields] AND "faso"[All Fields]) OR "burkina faso"[All Fields]) OR ("burundi"[MeSH Terms] OR "burundi"[All Fields]) OR ("cambodia"[MeSH Terms] OR "cambodia"[All Fields] OR "cambodia s"[All Fields]) OR ("central african republic"[MeSH Terms] OR ("central"[All Fields] AND "african"[All Fields] AND "republic"[All Fields]) OR "central african republic"[All Fields]) OR ("chad"[MeSH Terms] OR "chad"[All Fields]) OR ("comoros"[MeSH Terms] OR "comoros"[All Fields] OR "comoro"[All Fields]) OR ("democratic republic of the congo"[MeSH Terms] OR ("democratic"[All Fields] AND "republic"[All Fields] AND "congo"[All Fields]) OR "democratic republic of the congo"[All Fields]) OR ("djibouti"[MeSH Terms] OR "djibouti"[All Fields]) OR ("eritrea"[MeSH Terms] OR "eritrea"[All Fields]) OR ("ethiopia"[MeSH Terms] OR "ethiopia"[All Fields] OR "ethiopia s"[All Fields]) OR ("gambia"[MeSH Terms] OR "gambia"[All Fields] OR "gambia s"[All Fields]) OR ("guinea"[MeSH Terms] OR "guinea"[All Fields] OR "guinea s"[All Fields] OR "guineas"[All Fields]) OR ("guinea bissau"[MeSH Terms] OR "guinea bissau"[All Fields] OR ("guinea"[All Fields] AND "bissau"[All Fields]) OR "guinea bissau"[All Fields]) OR ("haiti"[MeSH Terms] OR "haiti"[All Fields] OR "haiti s"[All Fields]) OR ("micronesia"[MeSH Terms] OR "micronesia"[All Fields] OR "kiribati"[All Fields]) OR ("laos"[MeSH Terms] OR "laos"[All Fields] OR ("lao"[All Fields] AND "people s"[All Fields] AND "democratic"[All Fields] AND "republic"[All Fields]) OR "lao people s democratic republic"[All Fields]) OR ("lesotho"[MeSH Terms] OR "lesotho"[All Fields]) OR ("liberia"[MeSH Terms] OR "liberia"[All Fields] OR "liberia s"[All Fields]) OR ("madagascar"[MeSH Terms] OR "madagascar"[All Fields] OR "madagascar s"[All Fields]) OR ("malawi"[MeSH Terms] OR "malawi"[All Fields] OR "malawi s"[All Fields]) OR ("mali"[MeSH Terms] OR "mali"[All Fields]) OR ("mauritania"[MeSH Terms] OR "mauritania"[All Fields]) OR ("mozambique"[MeSH Terms] OR "mozambique"[All Fields] OR "mozambique s"[All Fields]) OR ("myanmar"[MeSH Terms] OR "myanmar"[All Fields] OR "myanmar s"[All Fields] OR "myanmars"[All Fields]) OR ("nepal"[MeSH Terms] OR "nepal"[All Fields] OR "nepal s"[All Fields]) OR ("niger"[MeSH Terms] OR "niger"[All Fields]) OR ("rwanda"[MeSH Terms] OR "rwanda"[All Fields] OR "rwanda s"[All Fields]) OR ("sao tome and principe"[MeSH Terms] OR ("sao"[All Fields] AND "tome"[All Fields] AND "principe"[All Fields]) OR "sao tome and principe"[All Fields]) OR ("senegal"[MeSH Terms] OR "senegal"[All Fields] OR "senegal s"[All Fields]) OR ("sierra leone"[MeSH Terms] OR ("sierra"[All Fields] AND "leone"[All Fields]) OR "sierra leone"[All Fields]) OR ("melanesia"[MeSH Terms] OR "melanesia"[All Fields] OR ("solomon"[All Fields] AND "islands"[All Fields]) OR "solomon islands"[All Fields]) OR ("somalia"[MeSH Terms] OR "somalia"[All Fields] OR "somalia s"[All Fields]) OR ("south sudan"[MeSH Terms] OR ("south"[All Fields] AND "sudan"[All Fields]) OR "south sudan"[All Fields]) OR ("sudan"[MeSH Terms] OR "sudan"[All Fields] OR "sudans"[All Fields] OR "sudan s"[All Fields]) OR ("tanzania"[MeSH Terms] OR "tanzania"[All Fields] OR "tanzania s"[All Fields]) OR ("timor leste"[MeSH Terms] OR "timor leste"[All Fields] OR ("timor"[All Fields] AND "leste"[All Fields]) OR "timor leste"[All Fields]) OR ("togo"[MeSH Terms] OR "togo"[All Fields]) OR ("micronesia"[MeSH Terms] OR "micronesia"[All Fields] OR "tuvalu"[All Fields]) OR ("uganda"[MeSH Terms] OR "uganda"[All Fields] OR "uganda s"[All Fields]) OR ("yemen"[MeSH Terms] OR "yemen"[All Fields]) OR ("zambia"[MeSH Terms] OR "zambia"[All Fields] OR "zambia s"[All Fields]))

Number of Articles: 114

1. **Database: Cochrane and Embase**

**Database search as of 12^th^ August 2023**

Search Term: Antimicrobial stewardship

Records: 118

Antimicrobial stewardship intervention in low income country

antimicrobial stewardship in Title Abstract Keyword AND low income in All Text AND least developed country in All Text AND antimicrobial resistance in All Text OR Antimicrobial stewardship protocol in Title, Abstract.
